# Performance comparison of streptavidin magnetic beads for epcam expressing cancer cell lines for circulating tumor cell (CTC) enrichment in a flow-through immunomagnetic system

**DOI:** 10.1371/journal.pone.0322375

**Published:** 2025-05-09

**Authors:** Peng Liu, Sitian He, Pieter Hart, Alloïse van Kleef, Stan Boxum, Anouk Mentink, Yongjun Wu, Leon W. M. M. Terstappen, Pascal Jonkheijm, Michiel Stevens

**Affiliations:** 1 Department of Medical Cell Biophysics, Techmed Center, Faculty of Science and Technology, University of Twente, Enschede, The Netherlands; 2 Department of Molecules and Materials, Laboratory of Biointerface Chemistry and the TechMed Centre, University of Twente, Enschede, The Netherlands; 3 College of Public Health, Zhengzhou University, Zhengzhou, China; 4 Department of General, Visceral and Pediatric Surgery, Heinrich-Heine University, University Hospital Düsseldorf, Düsseldorf, Germany; 5 FETCH BV, Deventer, The Netherlands; Hong Kong Metropolitan University, HONG KONG

## Abstract

Circulating tumor cells (CTCs) are important biomarkers for cancer diagnosis and treatment monitoring. However, their scarcity limits their utility as current enrichment techniques are hampered by low volume throughput and/or the inability to capture CTCs with low target antigen densities. Our group previously reported a device capable of processing samples in a flow manner using an optimized Halbach array to enhance the capture of low EpCAM-expressing cells (Flow-through Immunomagnetic CTC Enrichment system). In this study, we tested the capture efficiency of eight commercially available streptavidin magnetic beads using this device to identify the most suitable bead. Results indicate that using this system, the best-performing magnetic beads are in the ~ 100 to ~ 150 nm size range. Considering the combination of binding efficiency and final sample purity, we found that among the beads tested in combination with biotinylated anti-EpCAM, MojoSort Streptavidin Nanobeads performed the best, with high capture efficiencies for both the high EpCAM expressing LNCaP and low EpCAM expressing PC3–9 cell lines. For CTC enrichment from the blood of cancer patients, reducing the number of WBCs co-enriched with these beads will be essential, especially when processing large-volume samples acquired, for instance, through diagnostic leukapheresis to overcome the limitations caused by the scarcity of CTCs.

## Introduction

Circulating tumor cells (CTCs) are cancer cells that detach from the primary tumor and enter the bloodstream, thereby becoming potential “seeds” in the process of cancer metastasis [1]. Clinically, CTC enumeration and molecular characterization can help stratify patients by prognosis, guide targeted therapies, and monitor treatment response in real-time. For instance, changes in CTC counts can indicate disease progression or therapeutic efficacy sooner than traditional imaging, thus aiding in timely treatment adjustments. The isolation and detection of CTCs are crucial for advancing cancer diagnosis and treatment. However, the heterogeneity and rarity of CTCs make it challenging to isolate intact CTCs from blood samples of cancer patients while minimizing interference by co-isolated leukocytes [2,3]. Here, the heterogeneity of CTCs refers to the phenotypic changes that occur during cancer progression and response to therapy [4], while the rarity of CTCs refers to their extremely low abundance in the bloodstream, with only a few CTCs present among millions of white blood cells [5].

One commonly used biomarker for capturing CTCs is the Epithelial Cell Adhesion Molecule (EpCAM). EpCAM-based capture methods are inherently biased towards capturing CTCs with epithelial cell characteristics and are less effective in carcinomas with downregulated EpCAM expression, such as during epithelial-mesenchymal transition (EMT) [6,7]. Nevertheless, we chose EpCAM for this study due to its established clinical relevance, extensive validation, and widespread commercial availability of EpCAM-targeting reagents. Focusing on a single, well-characterized target allowed us to directly compare the performance of various streptavidin-coated beads, laying the groundwork for future expansions to include other CTC markers. Additionally, the heterogeneity within carcinomas may also be reflected in the EpCAM expression of the CTCs. Large differences in EpCAM expression on CTCs, both between and within patients, were previously shown, with their average expression being similar to that of medium to low EpCAM-expressing cell lines [8]. Over the years, researchers have developed a variety of approaches to improve EpCAM-based detection to capture this EpCAM low-expressing fraction of cells. For example, Wang et al. have explored the simultaneous use of epithelial markers, such as EpCAM, and mesenchymal markers, like N-cadherin, to capture a broader spectrum of CTCs [9]. By combining two markers, they achieved efficient capture of both mesenchymal and epithelial cells simultaneously. Moreover, some researchers have employed strategies to increase the interactions between the targeted cell surface and substrate to obtain high capture efficiency [10]. However, these strategies are based on isolating and counting CTCs from small volumes of blood samples (2–10 mL), which can result in false negatives and limit their use in clinical diagnosis and treatment monitoring [11].

A potential solution for the scarcity of CTCs is the use of diagnostic leukapheresis (DLA), which has emerged as a promising technique for enriching CTCs [11]. This method operates on the principle that peripheral mononuclear cells (MNCs) and CTCs have similar densities and can be collected by continuous flow centrifugation [12]. DLA can harvest CTCs from 1–5 liters of blood, and this considerably larger blood volume screened results in higher CTC yields, thus reducing the possibility of false negatives [13]. However, processing complete DLA samples presents an unsurmountable challenge for existing assays [12,14]. For example, the CellSearch method, which is currently widely accepted, encounters limitations in terms of high leukocyte concentrations in DLA samples [12,15]. Further development of more flexible separation systems that can accommodate different volumes and concentra-tions is needed in the pursuit of accurate and efficient CTC analysis.

To address these challenges, we previously developed an optimal Halbach configuration for a Flow-through Immuno-magnetic CTC Enrichment system (S1 Fig), which can process not only blood samples but also larger volume samples obtained through DLA [16]. Xue et al. also used a Halbach array for the continuous-flow separation of rare tumor cells [17]. Our system has been successfully tested using commercially available CellSearch immunomagnetic ferrofluids on various cell lines to optimize the Halbach array configuration and has also been shown to work using DLA samples obtained from hormone-sensitive prostate cancer patients [18]. For the optimized Halbach configuration, we achieved enhanced recovery of cell lines with low-level EpCAM antigen expression. However, until now, only the CellSearch ferrofluids, which are directly coupled to the EpCAM Antibody (clone VU1D9), have been tested on this system. It has been shown in the literature that the indirect capture method can improve capture efficiency compared to the direct capture method [19]. In this study, we aim to determine whether other beads are suitable for use in this system via the indirect capture method. For this reason, we characterized eight commercially available streptavidin-coated magnetic beads for the indirect capture of CTCs and evaluated their performance on this system. The magnetic bead sizes we selected range between 50 nm and 2.8 µm. These differences in bead size result from selecting commercially available beads specifically for CTC capture. Among them, nanoparticles smaller than 200 nm may enter cells due to internalization and cause false positive results. However, in our experiments, the selection of fixed cells greatly reduced the possibility of magnetic beads internalizing into cells, thereby minimizing false positives. For applications involving live cells, strategies such as optimizing the bead incubation time, utilizing blockers to prevent endocytosis, or verifying cell-surface binding via immunofluorescent staining could help minimize internalization. Additionally, post-enrichment validation methods (e.g., imaging, single-cell transcriptomics) can further distinguish true CTCs from potential false positives induced by intracellular bead uptake.

## Materials and methods

### Magnetic beads

Streptavidin beads were purchased from Miltenyi Biotec B.V. & Co. KG (Bergisch Gladbach, Germany). MojoSort Streptavidin Nanobeads were purchased from BioLegend (San Diego, CA, USA). FerroSelect Ferrofluids were purchased from BioMagnetic Solutions (State College, PA, USA). MagVigen Streptavidin was purchased from NVIGEN Inc. (Santa Clara, CA, USA). AccuNanoBead Magnetic Nanobeads were purchased from Bioneer Corporation (Oakland, CA, USA). Proteintech Streptavidin Magnetic Beads were purchased from Proteintech Group, Inc. (Rosemont, IL, USA). Dynabeads M-270 Streptavidin was purchased from Thermo Fisher Scientific (Waltham, MA, USA). MagnaLINK Streptavidin Magnetic Beads were purchased from TriLink Biotechnologies (San Diego, CA, USA).

### Flow-through immunomagnetic CTC enrichment system

The principle and characteristics of the flow-through immunomagnetic CTC enrichment system used were previously described [16]. In short, a shallow flow channel is positioned below an array of magnets that are assembled in a Halbach configuration resulting in the efficient extraction of magnetically labeled cells from the flow channel.

To perform the separation, after incubation with magnetic beads, the samples were flown through a 50 x 5 x 0.6 mm (L x W x H) Ibidi µ-Slide I Luer (Ibidi GmbH, Gräfelfing, Germany) channel. For this, a 10 mL syringe (BD Biosciences, San Jose, CA, USA) was placed on a programmable syringe pump (Harvard Apparatus) and connected to the sample outlet of the Ibidi µ-Slide channel via 1.0 mm inner diameter tubing. The same tubing was connected to the sample inlet of the flow chamber and the tubing’s other end was placed into the tube containing the sample. The samples were then aspirated through the Ibidi µ-Slide channel positioned directly under the Halbach magnet array at a flow rate of 0.5 mL/min. To rinse the leftover cells in the sample tube, 2 mL of PBS/1% BSA buffer was added to the sample tube once the samples had been almost aspirated completely. When the suspension was fully aspirated the pump was halted and after removal of the magnetic array the enriched fraction was collected using 2 mL PBS/1% BSA. The captured fractions were then magnetically washed 3 times with 1 mL PBS/1% BSA. “Magnetically washed” refers to a process where the magnetic bead-cell complex is placed on a magnet stand, and the supernatant is removed after five minutes. 1 mL PBS/1% BSA is then added to resuspend the complex, followed by repeating the magnetic separation step. Finally, the magnetic bead-cell complex is resuspended for flow cytometry measurement. This method ensures effective washing and isolation of the target cells.

**Fig 1:**
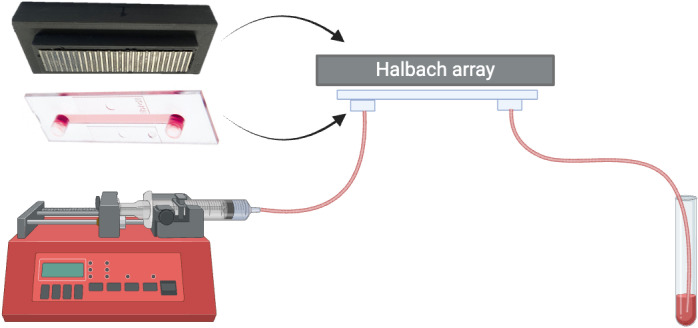
Schematic diagram of the flow-through immunomagnetic CTC enrichment system. This system consists of a µ-Slide channel with tubes connected to the inlet and outlet, underneath is a Halbach magnet array. Image created with BioRender.

The optimized Halbach array used consists of 57 N52 magnets, each with a length of 12 mm, a width of 1 mm, and a height of 2 (horizontal magnetization) or 2.75 mm (vertical magnetization) (Risheng Magnets, Ningbo, China). All arrays are assembled on soft magnetic sheets, and a 3D-printed plastic support is adhered to the back of the arrays. Subsequently, the soft magnetic material is removed, allowing the channels to directly contact the magnet surfaces (Fig 1). These configurations are consistent with those reported previously [16].

In our experiments, we used the Ibidi µ-Slide I Luer as the microfluidics channel. The dimensions of the microchannel in this µ-Slide are 50 mm in length, 5 mm in width, and 0.6 mm in height, resulting in a volume of 150 μL. This µ-Slide was designed for cell culture; we therefore choose to use the uncoated version to minimize cell binding to the channel surface.

### Cell lines

The Lymph Node Carcinoma of the Prostate (LNCaP) cell line was acquired from ATCC (Manassa, VA, USA), while the Prostate Cancer (PC3–9) cell line, a sub-clone of the PC3 cell line, was generously provided by Immunicon (Huntingdon Valley, PA, USA). Both PC3–9 and LNCaP cells were cultured in RPMI 1640 medium (Gibco, Waltham, MA, USA), supplemented with 10% Fetal Bovine Serum (FBS) (Sigma-Aldrich, St. Louis, MO, USA) and 1% Penicillin-Streptomycin Mixture (Lonza, Basel, Switzerland). The cells were maintained in a humidified atmosphere at 37 °C and trypsinized using 0.05% trypsin-EDTA (Gibco, Waltham, MA, USA) when they reached 70–80% confluence. All the cell lines were fixed by 1% formaldehyde after harvest. Fixation impacts the EpCAM expression on the cell line surface, as shown previously [8]. For instance, compared to unfixed LNCaP cells, the EpCAM expression of 1% formaldehyde-fixed LNCaP cells decreased by 71%. In this work, we have chosen to use fixated cells as our primary interest is in maintaining consistent cell characteristics throughout our extensive series of experiments.

To quantify the expression of the EpCAM antigen on the surface of PC3–9 and LNCaP cells, we employed phycoerythrin (PE)-conjugated VU1D9 (Sigma-Aldrich, St. Louis, MO, USA) for cell staining. Specifically, 10^5^ cells were resuspended in 80 μL of PBS/1% BSA, and then 20 μL of PE-conjugated VU1D9 was added. After incubation at 37°C for 30 min, the cells were washed twice and resuspended in 250 μL of PBS/1% BSA. Subsequently, the cell staining intensity was measured by flow cytometer (FACS Aria II, BD Biosciences, San Jose, CA, USA) and the number of PE molecules on each cell was quantified using the PE Fluorescent Quantitation Kit (BD Biosciences, San Jose, CA, USA).

### The characterization of magnetic beads

We characterized the morphology and zeta potential of eight commercially available magnetic beads using High Resolution-Scanning Electron Microscopy (HRSEM) (ZEISS Merlin, Oberkochen, Germany) and a Zetasizer Nano ZS (Malvern Panalytical, Malvern, United Kingdom). For HRSEM sample preparation, the beads were first washed twice with Milli Q Water to remove any salts from the solution. Subsequently, a droplet of the bead suspension was deposited onto the HRSEM sample platform and allowed to dry naturally at room temperature before morphology characterization. For zeta potential characterization, the recommended volume of beads was added to 1 mL 10-fold diluted phosphate-buffered saline (PBS) (Sigma-Aldrich St. Louis, MO, USA). Measurements were performed using a DTS1070 Zeta potential cell. We measured the zeta potential of magnetic beads in 10-fold diluted PBS to provide a controlled and reproducible environment, minimizing ionic interference for initial characterization. This standard method allows for baseline comparisons before evaluating particle behavior in more complex biological media, such as blood plasma [20,21].

### Healthy donor blood samples

Blood was collected from anonymized healthy volunteers in CellSave Preservative Tubes (Menarini Silicon Biosystems, Florence, Italy) at the University of Twente. In agreement with the Declaration of Helsinki, informed consent was obtained from all volunteers, and the blood collection procedure was approved by the local Medical Research Ethics Committee (METC Twente). All methods were carried out in accordance with relevant guidelines and regulations.

CellSave fixed whole blood samples (for instance, the reference sample, 1 mL) were lysed using BD FACS™ Lysing Solution 10X Concentrate (BD Biosciences, San Jose, CA, USA) diluted to 1X with MQ water before FACS measurement. For this, 10 mL of 1X lysing solution was added to each sample, followed by gentle inversion to mix. The samples were incubated at room temperature for 15 minutes and then centrifuged at 500 g for 5 minutes. The supernatant was discarded, and the cell pellet was washed once with PBS/1% BSA buffer through centrifugation.

### Capture performance of eight magnetic beads on cell line spiked samples

To compare the performance of the eight different magnetic beads for the capture of tumor cells from blood samples using our flow-through system, we first pre-stained 1% formaldehyde fixed LNCaP and PC3–9 cells using CellTracker Green and CellTracker Orange, respectively (Thermo Fisher Scientific, Waltham, MA, USA). Then, approximately 30,000 cells of each tumor cell line were spiked into 9 mL of whole blood from healthy donors. Next, biotinylated VU1D9 (unconjugated VU1D9 is a kind gift from Immunicon, Huntingdon Valley, PA, USA, biotinylated in-house) was added and the sample was incubated at 37 °C for 30 minutes. Following antibody incubation, the labeled blood was divided into nine 1 mL aliquots. Eight of these aliquots were incubated with different commercially available magnetic beads (using manufacturer-recommended volume, as shown in Table 1) for 30 minutes, where we used magnetic incubation for beads with sizes less than 200 nm and roller mixer incubation for beads sized more than 200 nm for better mixing.

**Table 1:** Manufacturer supplied or measured characteristics of the magnetic beads tested.

Beads	Size (manufacturer)	Size (measured by HRSEM)	Zeta potential (mV)	Morphology	Volume used for 1 mL	Package size	Price (USD)	Cost used volume for 1 mL blood (USD)
Miltenyi Streptavidin Microbeads	50 nm	64 ± 16	-0.11	Round dot	10 μL	For 1 × 10^9^ cells, up to 100 separations	348	3.48
MojoSort Streptavidin Nanobeads	130 nm	105 ± 38	-0.2	Irregular cluster	10 µ L	1 mL	330	3.30
BioMagnetic Solutions FerroSelect Ferrofluid	100-200 nm	133 ± 55	-0.08	Irregular cluster	3.3 µ L	5mL	2200	1.45
MagVigen Streptavidin	200-500 nm	237 ± 68	-25.29	Irregular cluster	20μL	2 mg/mL, 1 mL	232	4.64
AccuNanoBead Magnetic Nanobeads	400 nm	278 ± 37	-43.44	Cube	10 µ L	10 mg/mL, 5 mL	451	0.18
Proteintech Streptavidin Magnetic Beads	2.7 μm	2152 ± 42	-16.81	Sphere	2 µ L	10 mg/mL, 1 mL	250	0.50
Dynabeads M-270 Streptavidin	2.8 µm	2593 ± 102	0.025	Sphere	25 µ L	10 mg/mL, 2 mL	681	8.52
MagnaLINK Streptavidin Magnetic Beads	2.8 µm	2496 ± 207	0.22	Sphere	2 µ L	10 mg/mL, 1 mL	330	0.66

The ninth aliquot served as a reference for the number of white blood cells (WBCs) and spiked tumor cells and was used to determine recovery. After magnetic separation using the flow-through immunomagnetic CTC enrichment system, all captured cells as well as the reference sample were stained with Hoechst 33342 (Thermo Fisher Scientific, Waltham, MA, USA) for identification of nucleated cells and enumerated by flow cytometry. The capture efficiency was calculated as:


$$Capture\;efficiency\;(\;\%)=\;\frac{Captured\;cells}{Reference}\;\times 100\;\%,\;$$ (1)

Where “Captured cells” refers to either the number of white blood cells or tumor cells captured, and “Reference” refers to either the number of white blood cells or tumor cells enumerated in the references, depending on whether the specific capture efficiency for tumor cells or the non-specific capture efficiency for white blood cells is calculated.

The capture purity was calculated as:


$$Capture\;purity(\;\%)=\;\frac{Captured\;tumor\;cells}{All\;captured\;cells}\;\times 100\;\%$$ (2)

### Flow cytometry analysis

All the samples were measured on a flow cytometer using a 50 μm filter as a standard additional safety measure to prevent clogging. Unstained LNCaP cells and PC3–9 cells were used as negative control, and LNCaP stained using Hoechst and CellTracker green, as well as PC3–9 stained using Hoechst 33342 and CellTracker orange were used as positive controls. Thresholds for the forward scatter and the 375–450/40 channel were set to identify nucleated cells. The voltages of the channels detecting CellTracker Green and CellTracker Orange were adjusted to distinguish the stained cells and the unstained cells, and the gating strategy ensured that only the target cell populations were included in the gates. Compensation values were set to compensate for the spectral overlap between the fluorescence channels. Our enumeration method involved a process of weighing samples before and after FACS analysis. We weighed the empty FACS tubes before adding any sample, then we added the sample and weighed the tube and sample together to obtain the weight of the added sample. Lastly, after the FACS measurement, we weighed the leftover sample and the tube together, and calculated the consumed weight of the sample by subtracting it from the weight of the added sample before FACS measurement. This allowed us to accurately calculate the percentage of the sample analyzed, ensuring uniformity across different samples. In all cases at least 30% of the volume was measured. In cases where the cell concentration was low, the majority of the volume was measured in order to obtain sufficient cell events to achieve reliable results. By dividing the number of cell events detected by the fraction measured we calculated the total number of tumor and white blood cells in the sample. This allowed us to maintain consistency and comparability between samples.

### Statistics

We used one-way ANOVA to compare the capture efficiency of tumor cells between all eight magnetic beads. Origin 2023 SR0 (OriginLab Corporation, Northampton, MA, USA) was used to conduct all statistical and correlation analyses using a 0.05 significance threshold.

## Results

### Characterization of magnetic beads

**Fig 2:**
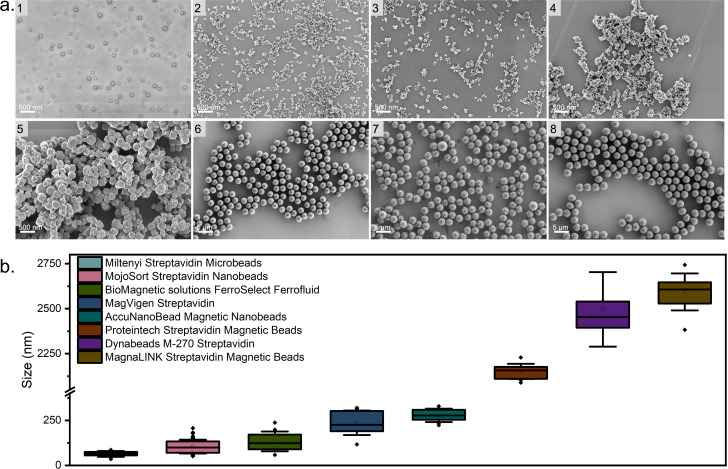
(a) HRSEM images of the eight types of beads. 1. Miltenyi Streptavidin Microbeads; 2. MojoSort Streptavidin Nanobeads; 3. BioMagnetic solutions FerroSelect Ferrofluid; 4. MagVigen Streptavidin; 5. AccuNanoBead Magnetic Nanobeads; 6. Proteintech Streptavidin Magnetic Beads; 7. Dynabeads M-270 Streptavidin; 8. MagnaLINK Streptavidin Magnetic Beads. (b) Size statistics from beads are shown in Panel a.

The size and surface area-to-volume ratio of magnetic beads are crucial in cell separation. Generally, the large, micrometer-sized beads have high magnetization and fast response speed, but low capture efficiency [22]. On the other hand, small-sized beads can achieve high capture efficiency but require longer separation times or higher magnetic gradients [23]. We investigated the morphologies and sizes of the commercial beads using HRSEM as shown in Fig 2a. We determined the bead sizes as shown in Fig 2b by ImageJ (National Institutes of Health, USA).

**Fig 3:**
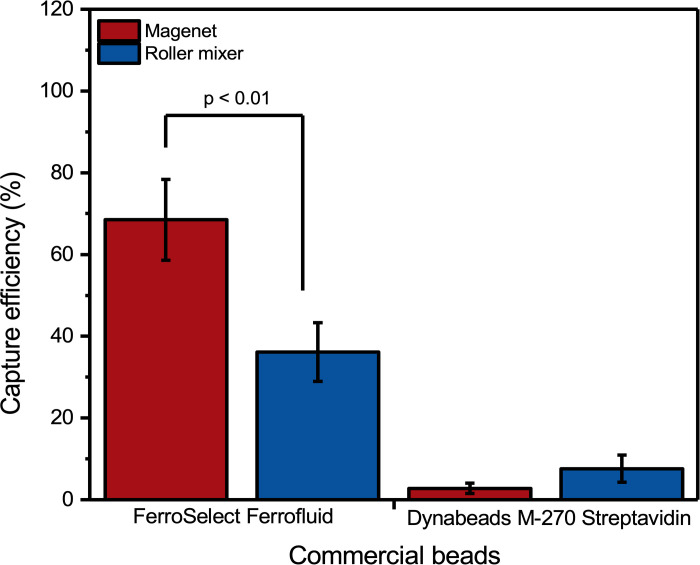
The impact of the incubation method on capture efficiency of Dynabeads M-270 Streptavidin beads and BioMagnetic Solutions FerroSelect Ferrofluid.

**Fig 4:**
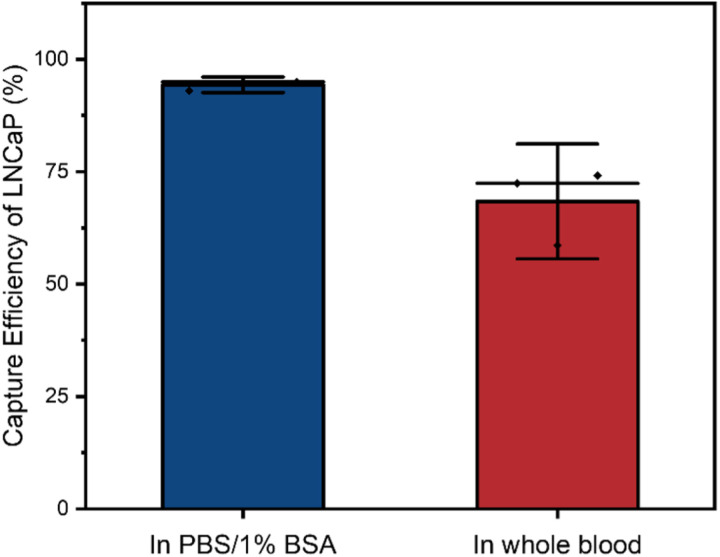
The capture efficiency of BioMagnetic solutions FerroSelect in PBS/1% BSA and whole blood showed a decrease in capture efficiency when moving to whole blood.

**Fig 5:**
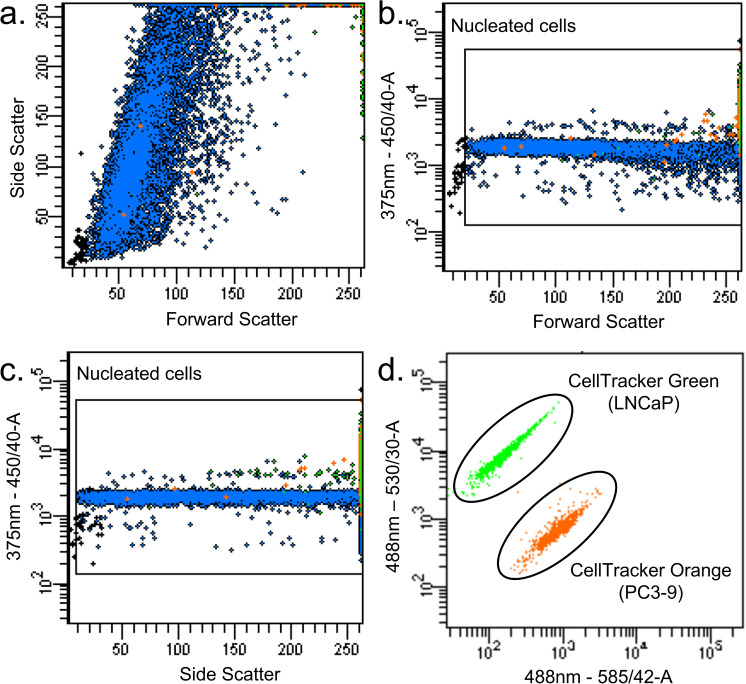
Example of a flow cytometer measurement. **(a)** Forward and Side Scatter of BioMagnetic Solutions FerroSelect Ferrofluid enriched LNCaP and PC3-9 cells from 1 mL of blood. The leukocytes depicted in blue increase their side scatter due to the presence of ferrofluids on the cell surface. (b) forward scatter versus Hoechst and panel (c) side scatter versus Hoechst with the gated used to identify nucleated cells. (d) excitation by the 488 nm laser and emission at 585 nm and 530 nm to distinguish the CellTracker Green colored LNCaP cells and the CellTracker Orange colored PC3-9 cells.

All measured sizes of the magnetic beads were found to be within the size ranges given by their respective manufacturers. In Fig 2a-1 the HRSEM image of Miltenyi Streptavidin Microbeads is shown, which were found to have a size of 60 nm. Fig 2a-2 to Fig 2a-4 display HRSEM images of MojoSort Streptavidin Nanobeads (105 ± 38 nm), BioMagnetic solutions FerroSelect Ferrofluid (133 ± 55 nm) and MagVigen Streptavidin (237 ± 68 nm). These three beads are similar-sized and all are shown to be nonuniform in structure. The AccuNanoBead Magnetic Nanobeads in Fig 2a-5 are 278 ± 37 nm-sized and both spherical and cubic nonuniform in morphology.

**Fig 6:**
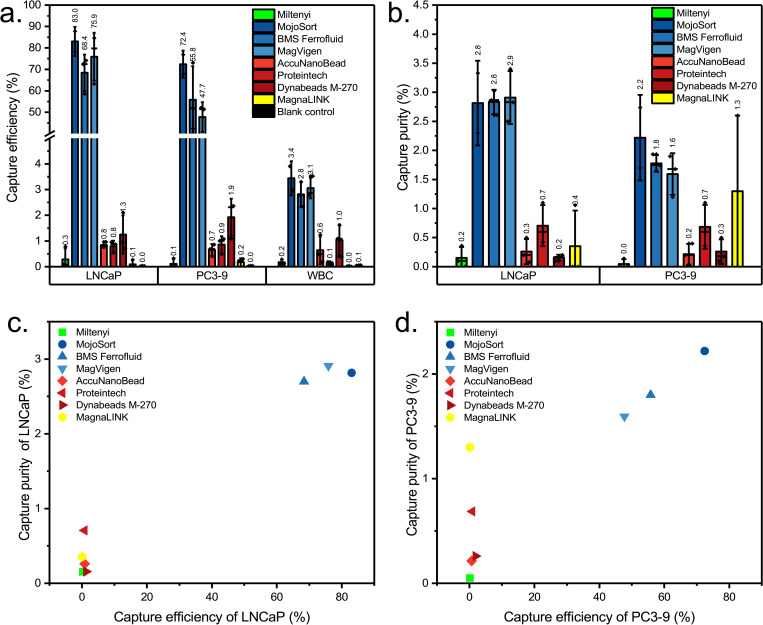
Capture efficiency and purity for all beads when capturing LNCaP & PC3-9 from blood. **(a)** The percentage of LNCaP, PC3-9, and WBC (leukocytes) captured. **(b)** The purity of the LNCaP & PC3-9 cells in the captured aliquots. Columns indicate mean ± SD with a median line. The capture efficiency versus purity is shown for LNCaP in panel (c) and PC3-9 in panel **(d)**.

The HRSEM images in Fig 2a-6 to Fig 2a-8, show spherical structures for Proteintech Streptavidin Magnetic Beads (2152 ± 42 nm in diameter), Dynabeads M-270 Streptavidin (2593 ± 102 nm in diameter), and MagnaLINK Streptavidin Magnetic Beads (2496 ± 207 nm in diameter).

Zeta potential can reflect the dispersion stability of colloids. Colloids with a high absolute value of zeta potential are electrically stable, while colloids with a low absolute zeta potential tend to coagulate or flocculate [24]. Generally, beads having zeta potentials between 0–5 mV are considered to coagulate easily, 10–30 mV are considered to be unstable, 30–40 mV are considered to be moderately stable, and greater than 40 mV indicates good stability [25]. We therefore measured the Zeta potential of each bead in ten-fold diluted PBS solution as shown in S2 Fig. However, we observed rapid sedimentation of AccuNanoBead Magnetic Nanobeads despite measuring the most negative zeta potential, indicating that zeta potential measured in 10X diluted PBS may not be a reliable predictor in this context. This could be due to the large size of the beads, resulting in significant gravitational force, or differences in surface modification, where the electrostatic repulsion force is insufficient to maintain their resuspension.

As in many laboratories, the costs of the used reagents including magnetic beads are an important consideration, and we calculated the cost according to the used volume for processing 1 mL of blood for each bead. The cost per test shows a 47-fold difference between the lowest and highest cost, with prices ranging from 0.18 USD to 8.52 USD. The volumes used were based on the manufacturer’s recommendations for bead usage relative to cell numbers or volumes. This approach provides a perspective on the economic feasibility of using different beads in clinical and research settings, aiding in selecting the most cost-effective option for specific applications. In Table 1 the given size, measured size, recommended usage volume, package size, and price, as well as the cost of the used volume to process 1 mL of blood, are listed.

### Feasibility of the assay

In immunomagnetic separation, the incubation method is also critical, as it significantly influences the binding efficiency between the magnetic beads and the target cells. In line with the most prominent immunomagnetic CTC enrichment technology, CellSearch, which uses approximately 200 nm-sized magnetic beads, we have tested the use of magnetic incubation [26]. However as some of the beads used are attracted to the magnets within seconds, we have also tested mixing the samples by agitation through the roller mixer [27]. The eight different types of beads in this study can be roughly categorized into nano-sized and micro-sized beads based on their measured sizes. The method of magnetic incubation involves using magnets to attract beads that travel across the tube to move toward the magnets. During their movements, they gain more chances to contact cells, which increases their opportunities to bind to the surface of target cells, and this is beneficial for improving the separation efficiency. This method was particularly effective for beads with sizes less than 200 nm. We utilized a roller mixer incubation method for micro-sized beads, which facilitated better mixing and interaction of beads with the sample due to their larger size and stronger magnetic properties.

To select the best incubation method and maximize the beads’ binding efficiency, we have chosen two representative beads from each size category, BioMagnetic Solutions FerroSelect Ferrofluid and Dynabeads M-270 Streptavidin, to explore the impact of the selected incubation method on capture efficiency.

For this experiment, we spiked approximately 3,600 LNCaP cells into a 1 mL blood sample from a healthy donor. We then incubated the sample using either a magnetic incubation method or a roller mixer, replicating the process three times for each type of bead used. The capture efficiency for Dynabeads M-270 Streptavidin beads increased from 2.8% for magnetic incubation to 7.6% for the roller mixer, while the capture efficiency of BioMagnetic Solutions FerroSelect Ferrofluid decreased from 69% with magnetic incubation to 36% for the roller mixer (Fig 3). This significant difference (t-test, p < 0.01) can be attributed to the relatively small BioMagnetic Solutions FerroSelect Ferrofluid that sediments much slower compared to the larger Dynabeads. Specifically, Dynabeads settle in minutes while FerroSelect Ferrofluid remains colloidal for several hours. The magnetic incubation in this case helps them to move, thereby increasing their chances of contact with target cells. Dynabeads M-270 Streptavidin beads, however, due to their larger size and strong magnetism, can be attracted to the magnet in tens of seconds, resulting in a very limited time window to bind to the target cells. To achieve the best result possible with all different beads, when comparing the capture efficiency of different beads in subsequent experiments, we used magnet incubation for nano-sized beads and roller mixer incubation for the micro-sized beads.

Another factor of importance is the sample medium, as the capture efficiency of tumor cells spiked in PBS, lysed blood or whole blood can be quite different as has been reported previously [19]. Using BioMagnetic Solutions FerroSelect Ferrofluid, we compared the capture efficiency of LNCaP cells spiked in PBS containing 1% BSA and whole blood (Fig 4). When using magnetic incubation and performing magnetic separation with the flow-through system, the capture efficiency of LNCaP, as shown in Fig 3, was 95% in PBS/1% BSA and only 68% in whole blood.

This difference can be attributed to the presence of red blood cells which make up 40–45% of the sample volume in the whole blood of healthy donors and hamper both the labeling as well as the movement of the magnetically labeled cells during the magnetic separation [28]. In addition, the presence of white blood cells, platelets, and plasma components can impair magnetic separation. For the white blood cells, the main problem is not that they impair tumor cell capture, but the non-specific binding of magnetic beads to the white blood cells, causing them to be co-enriched with the target cells. Considering that blood is the most common liquid biopsy sample drawn from cancer patients, we have chosen to spike blood samples with cells derived from tumor cell lines to allow a more realistic evaluation of the capture efficiency of the different magnetic beads.

### Capture capacity of eight magnetic beads in spiked blood samples on the flow-through immunomagnetic CTC enrichment system

Tumor cells are heterogeneous and have different EpCAM expression levels. This EpCAM expression density highly affects the capture efficiency when using different cell lines. We have therefore determined the EpCAM density of PC3–9 and LNCaP, showing an expression of 19,700 and 628,400 epitopes per cell, respectively. This indicates we can use these cell lines to represent CTCs with different EpCAM expression levels for the comparison of the eight commercial beads.

To distinguish PC3–9 and LNCaP in the spiked blood samples, we stained them with CellTracker Orange and CellTracker Green, respectively. To distinguish nucleated cells from other (debris) components in the sample, we stained them with Hoechst 33342. The flow cytometer plots in Fig 5 show the gating process of the captured portion in a sample enriched using the BioMagnetic Solutions FerroSelect Ferrofluid. Nucleated cells were defined as having a forward and side scatter in the appropriate range together with a positive Hoechst signal. LNCaP cells were defined as CellTracker Green positive cells falling within the nucleated cell gate, and PC3–9 cells as CellTracker Orange positive falling within the nucleated cell gate. All other nucleated cells were counted as leukocytes. When we used FACS to compare the performance of 8 types of magnetic beads, we chose a 50 μm filter. Its use will not affect the cell concentration and counting of pure cell-type samples.

Fig 6a shows the capture efficiency of LNCaP and PC3–9 as well as the percentage of nonspecifically captured WBCs. The MojoSort Streptavidin Nanobeads, BioMagnetic solutions FerroSelect Ferrofluid, and MagVigen Streptavidin have the highest capture efficiencies, ranging from 57% to 88% for LNCaP cells and from 40% to 77% for PC3–9 cells. and the non-specific capture percentages for leukocytes in Fig 6a are all below 3.5%. To compare the beads performance in both specific and non-specific capture, we have plotted the capture purity for each beads as shown in Fig 6b. Also in this regard the same three magnetic beads showed clear specific enrichment. Before enrichment, the purity of spiked tumor cells was approximately 0.1%, calculated as the number of spiked-in tumor cells (mean 3,200) divided by the number of WBCs in the 1 mL blood used as a reference (mean 3.1 × 10^6^ WBCs. After the enrichment, the purity of LNCaP cells was 2.8%, 2.8%, and 2.9% for MojoSort Streptavidin Nanobeads, BioMagnetic Solutions FerroSelect Ferrofluid, and MagVigen Streptavidin, respectively; the enrichment factors can be calculated as 28, 28, 29. The purity of PC3–9 cells was 2.2%, 1.8%, and 1.6% for MojoSort Streptavidin Nanobeads, BioMagnetic Solutions FerroSelect Ferrofluid, and MagVigen Streptavidin, respectively, with enrichment factors of 22, 18, 16, indicating the effectiveness of the enrichment. To show the difference between the non-specific binding caused by the beads and that of the chamber we added a negative control in which spiked blood was passed without the addition of beads, resulting in a non-specific capture of 0.1%. We have listed the overall capture efficiency and purity in Supplementary Table S1.

Depending on the exact application, there will be a higher demand for capture efficiency or purity, but in most cases, we must consider both when choosing beads for any application. To make this combined consideration visible, we have combined both features in Fig 6c and 6d.

**Fig 7:**
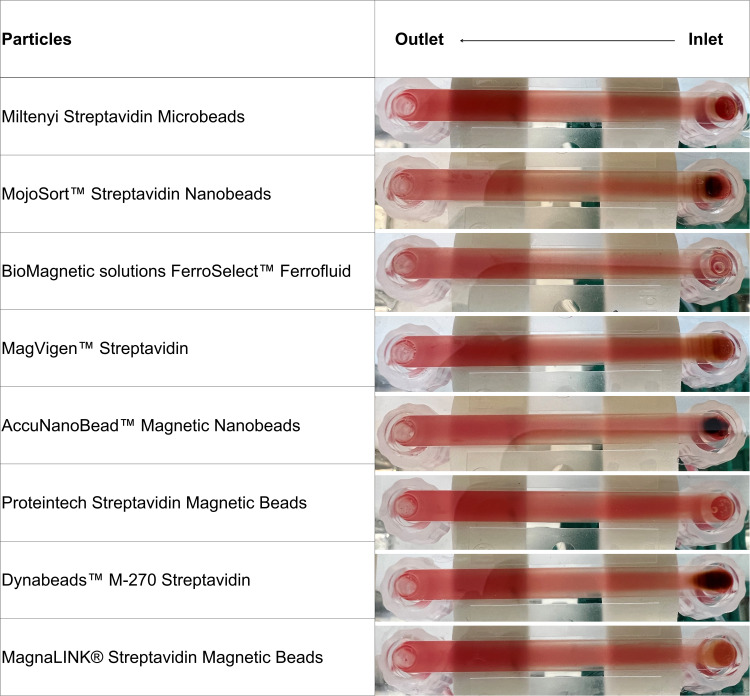
Photos of the different magnetic beads in the flow-through immunomagnetic CTC enrichment system.

Here, the more top-right the data point is, the better the overall performance. From the same plot, we observe clearly that MojoSort Streptavidin Nanobeads, BioMagnetic solutions FerroSelect Ferrofluid, and MagVigen Streptavidin stand out. These three beads are all sized between 100 nm to 500 nm, and the higher capture efficiency could be attributed to their higher surface area and more available biotin binding sites. The low capture efficiency of Miltenyi Streptavidin Microbeads might be caused by their small size and weak magnetization, indicating there may not be sufficient magnetic force generated for them to be attracted when flowing through the channel. As for AccuNanoBead Magnetic Nanobeads, as their size is around 278 nm, they would also be expected to show a high capture efficiency, but their use resulted a less than 1% capture efficiency. The reason for this could be aggregation, as we observed sedimentation in the bottle and during the experiment, and the HRSEM in Fig 2a-5 also shows bead clumping. Aggregation can mask active binding sites and alter the effective size distribution, thereby reducing overall capture efficiency and contributing to lower purity. Strategies such as sonication, addition of surfactants, or optimized buffer conditions may help minimize bead aggregation and improve CTC capture performance. In Fig 7 images of the flow channel after separations using the eight beads are shown. The dark color observed near the inlet in Fig 7 is primarily due to the accumulation of magnetic beads in this area. The shadow effect seen is a result of the lighting and imaging conditions used during the capture of this figure. The concentration of beads near the inlet is attributable to the magnetic field gradient created by our Halbach array setup. This observation is consistent with the magnetic properties of the beads and the fluid dynamics within the microchannel.

Here, the difference in the amount of beads used is visible. The more beads used, the larger the area of beads aggregated in the channel. In addition, the distribution of the captured beads along the Halbach array showed differences. The higher the magnetization, the more the beads were attracted close to the inlet where they formed clusters, while the lower the magnetization, the more the beads were distributed along the magnet array.

## Discussion

The presence of cancer cells in blood strongly suggests the presence of cancer [29,30]. Isolation of these CTCs and subsequent molecular characterization can be used to prove the presence of cancer, likely also in patients in which cancer has already spread to distant sites even though this does not yet manifest clinically [31]. The number of CTCs detected by current technologies such as the FDA-cleared CellSearch system is not sufficient for implementation in clinical practice for the detection of occult metastasis in patients with primary cancer. In patients with metastatic disease, monitoring of CTC load is being used to gauge the effect of treatment albeit in approximately 50% of the patients the number of CTCs is too low to accurately determine the disease status [32]. Increasing the examined blood volume and broadening of the target antigens will likely be needed to overcome this hurdle. Although we used EpCAM as the target antigen for CTC capture, the labeling of blood cells with biotinylated monoclonal antibodies in combination with streptavidin magnetic beads does not account for CTCs with low or absent EpCAM expression, such as those undergoing EMT. Consequently, this approach may miss certain subpopulations of CTCs with mesenchymal features. In future applications, expanding the marker panel (e.g., mesenchymal, tumor-specific, or tissue-specific antigens) will improve capture efficiency across diverse CTC phenotypes. The Flow-through Immunomagnetic CTC Enrichment system enables one to fully explore the potential of CTC detection by providing the flexibility to evaluate different target antigens and the ability to probe larger blood volumes to increase the sensitivity of CTC detection.

This flexibility in target antigen allows for the use of additional targets such as recombinant VAR2CSA (rVAR2) [33], prostate-specific membrane antigen (PSMA) [34], and Her2 [35]. These assays can be optimized using cell lines that express different levels of these antigens before starting evaluation in primary and metastatic cancer patients [8]. In this manuscript, we have used eight streptavidin-coated beads that are commercially available and recommended by the manufacturer for CTC capture. We used flow cytometry to count cells enriched with 8 magnetic beads and employed BD FACSAria filters (50 µm pore size) for clog-prevention. In this study, we primarily worked with single-cell suspensions, so the filter had minimal impact on cell enumeration. However, in clinical samples that may contain CTC clusters larger than 50 µm, these filters could lead to partial loss of such clusters, thus underestimating total CTC burden. Further work employing cluster-permissive protocols would be beneficial when studying the biological significance of CTC clusters. Additionally, the dense nature of these beads affects the signal amplitude and fluorescence, potentially leading to quantitative bias. As however this is in our approach part of the performances of the magnetic beads, it is also considered in the comparison of the final results. To minimize result variability in this concept comparison experiment, we chose a higher concentration of spiked cells (~30,000 per 9 mL) in our experiment than typical CTC levels found in patient samples (often <100 CTCs in 7.5 mL). Using larger spiking numbers ensures reproducibility and sufficient cell recovery to compare bead performance. Nonetheless, in real clinical scenarios, CTCs are far rarer, which makes high-volume or leukapheresis-based processing advantageous for capturing these scarce cells. Our findings here, under standardized conditions, guide the selection of suitable beads for more realistic, lower-CTC contexts. Although our study used fixed cells for consistent results across experiments, the usage of live cells would be beneficial for certain applications, such as downstream RNA or secretome analysis. The use of this novel separation system for the enrichment of CTCs from patient samples has recently been shown using DLA samples from hormone-sensitive prostate cancer patients [18].

There are however many more beads commercially available, some of which fall into the size range that appears to be most suitable for this separation system. Additionally, we have used here the recommended volume of MojoSort Streptavidin Nanobeads, BioMagnetic solutions FerroSelect Ferrofluid, and MagVigen Streptavidin, which might not be the optimal concentration for CTC capture in our separation system. Zeta potential affects the stability of magnetic beads, but in our experiments, we found that zeta potential and capture performance do not show a correlation. For example, as shown in Fig 5c and 5d, MojoSort Streptavidin Nanobeads, BioMagnetic solutions FerroSelect Ferrofluid and MagVigen Streptavidin are the three best-performing magnetic beads, but their zeta potentials are not consistent, with MojoSort Streptavidin Nanobeads and BioMagnetic solutions FerroSelect Ferrofluid having a zeta potential around 0 mV and MagVigen Streptavidin having a zeta potential around -20 mV. Considering long-term stability/storage, the zeta potential indicates it may be more advantageous to choose MagVigen Streptavidin compared to MojoSort Streptavidin Nanobeads and BioMagnetic solutions FerroSelect Ferrofluid.

For CTC enrichment from the 1 mL blood aliquots used here, the non-specific binding of leukocytes—which resulted in a capture purity of around 3%—does not significantly impede the detection of spiked tumor cells. However, when larger blood volumes or diagnostic leukapheresis (DLA) products are processed (e.g., 40 mL of blood or a 200 million WBC DLA product), the co-enrichment of even a small fraction of leukocytes can yield millions of contaminating cells, making it challenging to isolate rare CTCs [36,37]. Specifically, our experiments showed that the proportion of leukocytes non-specifically captured by streptavidin beads remained below 4% overall, consistent with some studies employing specialized materials [36,37]. Nonetheless, this level of leukocyte co-capture can still hamper the identification and isolation of tumor cells, especially when only a handful of CTCs are present [38].

Addressing this issue in clinical applications may require additional steps to lower non-specific binding and raise CTC purity, such as as combining methods, using for instance CD45 + cell depletion or microfluidic size or density based separation either before or after magnetic enrichment. Although these measures can increase both processing time and complexity, they are likely to enhance sample quality significantly—an especially pertinent factor for downstream molecular assays such as single-cell RNA sequencing. Likewise, adjusting bead concentrations, optimizing incubation protocols, or using beads designed to cluster selectively around target cells (e.g., in the CellSearch system) [26] could further diminish leukocyte contamination. Another potential approach is to move beyond EpCAM alone, utilizing alternative antigens to prevent the binding of any leukocytes that may express low levels of EpCAM-like epitopes.

## Conclusions

We evaluated the performance of eight different commercial streptavidin magnetic beads to capture cell line-derived tumor cells in blood labeled with EpCAM biotin monoclonal antibodies on our previously developed Flow-through Immunomagnetic CTC Enrichment system using 1 mL blood aliquots. From our evaluation, we concluded that MojoSort Streptavidin Nanobeads performed best considering capture efficiency, purity, and costs.

## Supporting information

S1 Fig Schematic representation of the magnetophoretic flow separation setup comprised of a flow channel with a Halbach array showing the magnetic (F_m_), drag (F_d_) and gravitational force (F_g_). (PDF)

S2 Fig The zeta potential of the beads. (PDF)

S3 Fig FSC/SSC plot of blank control. (PDF)

S1 Table The overall of capture efficiency and purity. (PDF)
