# Supplementary material for: Performance comparison of streptavidin magnetic beads for epcam expressing cancer cell lines for circulating tumor cell (CTC) enrichment in a flow-through immunomagnetic system
Source: PLoS One. 2025 May 9;20(5):e0322375. doi: 10.1371/journal.pone.0322375 (PMC12063838; doi:10.1371/journal.pone.0322375)
Supplement: S1 Fig — (PDF) [file pone.0322375.s001.pdf]

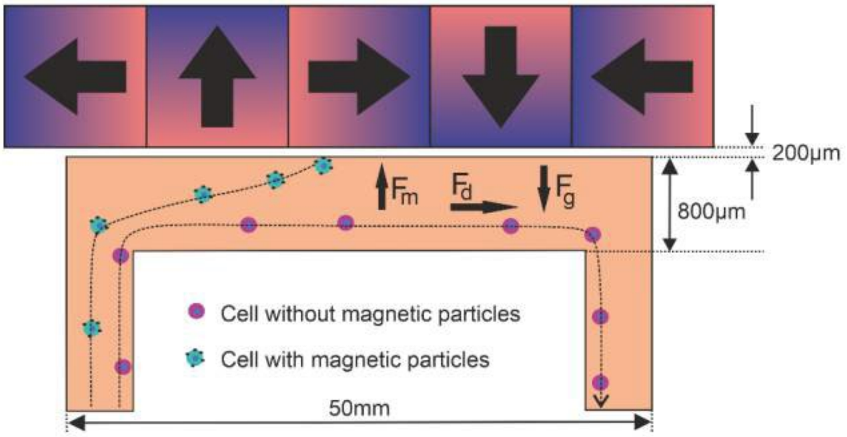

Figure S1. Schematic representation of the magnetophoretic flow separation setup comprised of a flow channel with a Halbach array showing the magnetic (F_m), drag (F_d) and gravitational force (F_g) (1).

References

1. Stevens M, Liu P, Niessink T, Mentink A, Abelmann L, Terstappen L. Optimal halbach configuration for flow-through immunomagnetic ctc enrichment. Diagnostics [Internet]. 2021 Jun 2 [cited 2024 Apr 6];11(6):1020. Available from: https://www.mdpi.com/2075-4418/11/6/1020/htm
