# Supplementary figures and images for: Performance comparison of streptavidin magnetic beads for epcam expressing cancer cell lines for circulating tumor cell (CTC) enrichment in a flow-through immunomagnetic system
Source: PLoS One. 2025 May 9;20(5):e0322375. doi: 10.1371/journal.pone.0322375 (PMC12063838; doi:10.1371/journal.pone.0322375)

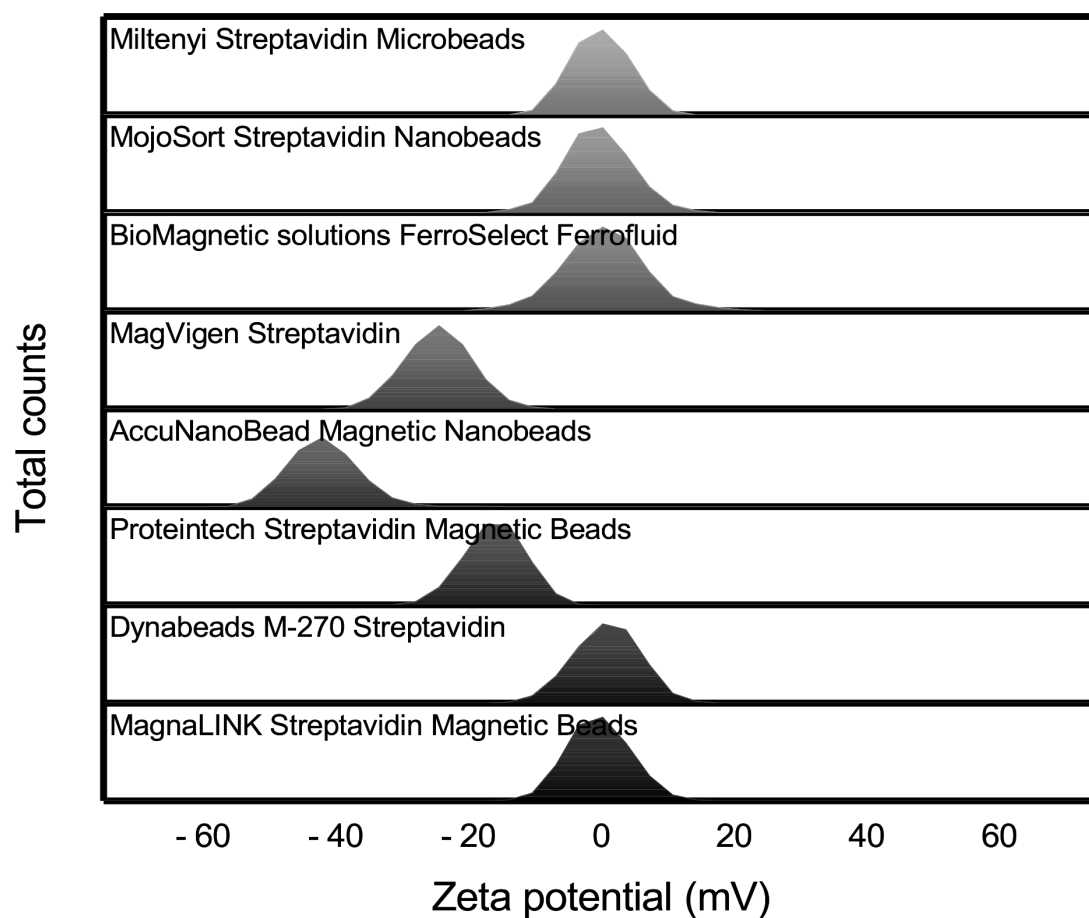

**Figure S2. The zeta potential of the beads.**

1  
2  
3

Supplement: S2 Fig — (PDF) [file pone.0322375.s002.pdf]

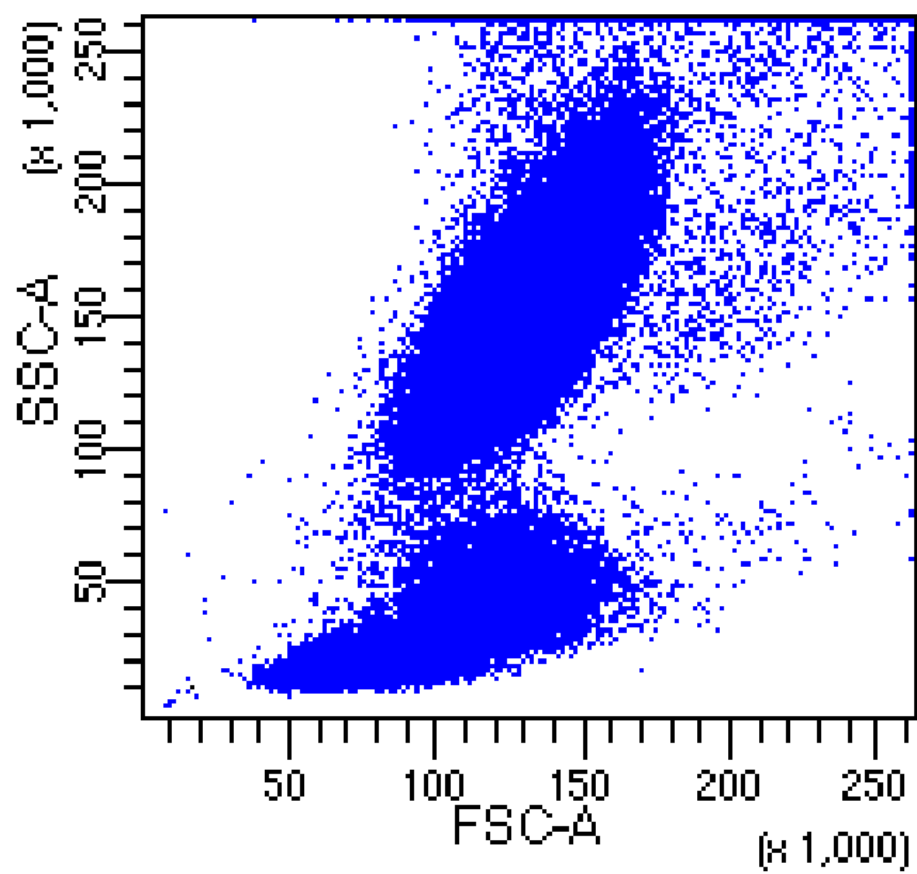

**Figure S3. FSC/SSC plot of blank control.**

1  
2  
3  
4  
5

Supplement: S3 Fig — (PDF) [file pone.0322375.s003.pdf]
