# Supplementary material for: Performance comparison of streptavidin magnetic beads for epcam expressing cancer cell lines for circulating tumor cell (CTC) enrichment in a flow-through immunomagnetic system
Source: PLoS One. 2025 May 9;20(5):e0322375. doi: 10.1371/journal.pone.0322375 (PMC12063838; doi:10.1371/journal.pone.0322375)
Supplement: S1 Table — (PDF) [file pone.0322375.s004.pdf]

Table S1. The overall of capture efficiency and purity.

Beads	Capture efficiency (%)			Capture purity (%)	
Cell line	LNCaP	PC3-9	WBC	LNCaP	PC3-9
Miltenyi Streptavidin Microbeads	0.3	0.1	0.2	0.2	0.0
MojoSort Streptavidin Nanobeads	83.0	72.4	3.4	2.8	2.2
BioMagnetic solutions FerroSelect Ferrofluid	68.4	55.8	2.8	2.8	1.8
MagVigen Streptavidin	75.9	47.7	3.1	2.9	1.6
AccuNanoBead Magnetic Nanobeads	0.8	0.7	0.6	0.3	0.2
Proteintech Streptavidin Magnetic Beads	0.8	0.9	0.1	0.7	0.7
Dynabeads M-270 Streptavidin	1.3	1.9	1.0	0.2	0.3
MagnaLINK Streptavidin Magnetic Beads	0.1	0.2	0.0	0.4	1.3
